# Quercetin and Ferroptosis

**DOI:** 10.3390/life13081730

**Published:** 2023-08-11

**Authors:** Alfredo Cruz-Gregorio, Ana Karina Aranda-Rivera

**Affiliations:** 1 Departamento de Biomedicina Cardiovascular, Instituto Nacional de Cardiología Ignacio Chávez, Mexico City 14080, Mexico; 2 Laboratorio F-315, Departamento de Biología, Facultad de Química, Universidad Nacional Autónoma de México, Mexico City 04510, Mexico; anitaaranda023@comunidad.unam.mx

**Keywords:** quercetin, ferroptosis, cancer, renal injury, liver injury, inflammation

## Abstract

Quercetin is a flavonoid present in apples, onions, tea, red wines, and berries, and it has shown different beneficial effects, such as providing cardiovascular protection, possessing anti-inflammatory properties, and demonstrating anticancer activity, among others. These diseases are related to oxidizing molecules such as ROS because these species react and induce the oxidation of cellular biomolecules, such as proteins, lipids, DNA, or carbohydrates, which alters cellular homeostasis. Regarding lipids, the oxidation of these molecules induces lipid hydroperoxides which, if not decreased, particularly by GPX4, produce highly reactive aldehydes such as 4HNE and MDA. These oxidative conditions induce ferroptosis, a type of cell death associated with oxidation that differs from other types of cell death, such as apoptosis, necrosis, or autophagy. The induction of ferroptosis is desired in some diseases, such as cancer, but in others, such as cardiovascular diseases, this type of cell death is not wanted. The possible effects of quercetin associated with reducing or inducing ferroptosis have not been reviewed. Thus, this review focuses on the ability of quercetin to produce ferroptosis in diseases such as cancer as a treatment option and, conversely, on its role in deactivating ferroptosis to alleviate diseases such as cardiovascular diseases.

## 1. Introduction

Plants synthesize and accumulate a plethora of natural products such as alkaloids, flavonoids, and terpenoids. These compounds serve as messengers in different cell signaling pathways, e.g., as signals for nitrogen-fixing bacteria; they are also protective agents since they act as shields against ultraviolet light, attractants for pollination and oviposition, and even antimicrobial/antiviral agents [1]. Flavonoids are polyphenolic compounds found in fruits, vegetables, and cereals that have numerous actions, such as antioxidant activities, inducing phase II metabolizing enzymes via the activation of Nrf2, controlling cellular growth, and antiviral and antibacterial actions. Interestingly, these molecules are more effective antioxidants in vitro than vitamins E and C and have lower levels of toxicity [2]. Quercetin is a flavonoid characterized by its flavone nucleus, which is composed of two benzene rings linked with a heterocyclic pyrone ring. It is found in apples, onions, tea, red wines, and berries. Quercetin has different beneficial effects, such as providing cardiovascular protection, possessing anti-inflammatory properties, anticancer activity, and antiulcer effects, and demonstrating antiallergic and antiviral actions [3]. The diseases represented by the latter effects are related to oxidant molecules such as reactive oxygen species (ROS) because these species react and induce the oxidation of cell biomolecules, such as proteins, lipids, DNA, or carbohydrates, which impair cell homeostasis [4]. Regarding lipids, the oxidation of these molecules induces lipid hydroperoxides, which glutathione peroxidase 4 (GPX4) degrades; however, if GPX4 reduces its expression or activity, highly reactive aldehydes such as 4-Hydroxynonenal (4HNE) and malondialdehyde (MDA) are produced. These oxidative conditions induce a type of cell death associated with oxidation, ferroptosis, which differs from other cell deaths such as apoptosis, necrosis, or autophagy [5]. The induction of ferroptosis is desired in some diseases, such as cancer, but in others, such as cardiovascular diseases, it is not. Quercetin has antioxidant and chelating qualities to conquer oxidation cell conditions and ferroptosis; in comparison with clinical chemotherapy treatments that are prone to serious side effects, quercetin was proven to be safe and effective as a potential anticancer compound with no significant adverse effects [6]. However, the possible effects of quercetin associated with reducing or inducing ferroptosis have not been reviewed. Thus, this review focuses on the ability of quercetin to produce ferroptosis in diseases such as cancer as a treatment option and, conversely, on its role in deactivating ferroptosis to alleviate diseases such as cardiovascular diseases.

## 2. Ferroptosis

Ferroptosis was described by Dixon et al. [5], who found that cysteine absorption decreased due to the presence of erastin, resulting in a decrease in glutathione (GSH), the accumulation of ROS and lipid peroxides, iron augmentation, decreases in the size of mitochondria, and increases in plasma membrane density with a decrease in or the disappearance of mitochondrial cristae and the induction of membrane rupture. The latter occurs because the increase in the amount of lipid peroxides destroys membrane integrity and alters membrane fluidity. The accumulation of lipid peroxides also induces the production of MDA and 4HNE, which form adducts with proteins, DNA, and phospholipids. All these processes induce oxidative cell death, which occurs without the typical characteristics of apoptosis, such as cell shrinkage, nuclear fragmentation, and the formation of apoptotic bodies; there are also no necrotic characteristics, such as cytoplasm swelling, nor does it share characteristics with autophagy, such as the formation of membrane-encapsulated vesicles [5]. In general terms, ferroptosis is a form of programmed cell death characterized by the accumulation of iron and the generation of ROS, involving the dysregulation of cellular iron metabolism, lipid peroxidation, and the depletion of antioxidant systems. It has been studied in various diseases, including cancer, neurodegeneration, diabetes, and renal and liver injuries, among others. Understanding the mechanisms and regulation of ferroptosis could be a potential tool in developing new therapeutic strategies for treating diseases associated with this type of cell death. Moreover, the induction of ferroptosis could provide new avenues to induce cell death in diseases such as cancer.

## 3. Quercetin Alleviates Ferroptosis in Renal Injury

**Figure 1:**
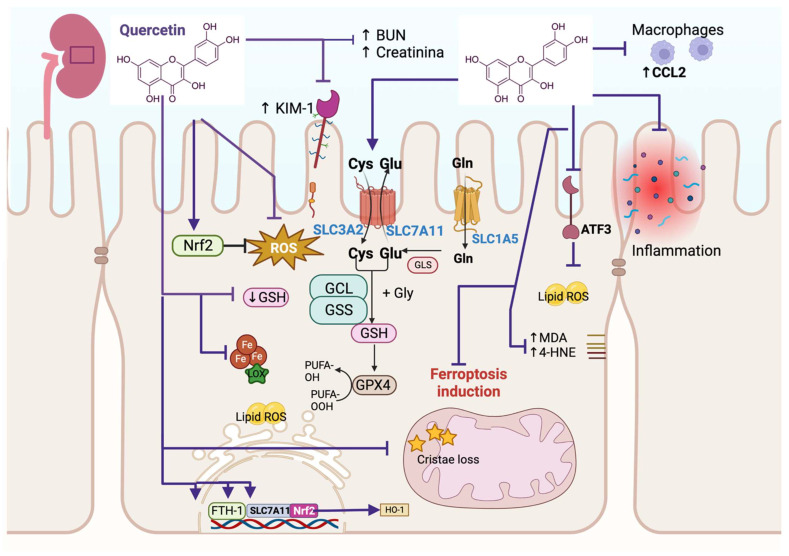
Quercetin alleviates ferroptosis in kidney damage. Quercetin prevents the increase in blood nitrogen urea (↑ BUN), creatinine, kidney injury molecule-1 (↑ KIM-1), and reactive oxygen species (ROS). It increases nuclear factor erythroid 2-related factor (Nrf2) solute carrier family 3 member 2 (SLC3A2) and solute carrier family 7 member 11 (SLC7A11), increasing glutathione (GSH), which promotes glutathione peroxidase 4 activity (GPX4). Quercetin prevents cristae loss in the mitochondria and induces the upregulation of the transcription factors iron storage protein ferritin heavy chain 1 (FTH-1), SLC7A11, and Nrf2. The upregulation of Nrf2 induces the transcription of heme oxygenase-1 (HO-1). Quercetin blocks malondialdehyde (↑ MDA) and 4-hydroxynoneal (↑ 4-HNE) and the activating transcription factor 3 (ATF3), preventing lipid ROS. These mechanisms prevent ferroptosis induction. Quercetin also avoids inflammation by downregulating chemokine (C-C motif) ligand 2 (CCL2), preventing macrophage recruitment. Cys: cysteine; Glu: glutamate; GCL: glutamate cysteine ligase; GSS: glutamate cysteine synthetase; PUFA: polyunsaturated fatty acid.

Fang et al. [7] showed that high-glucose (HG) treatment induces cell injury in human kidney proximal tubular epithelial (HK-2) cells and that quercetin alleviated this injury, increasing cell viability. Ferrostatin-1 (Fer-1), a ferroptosis inhibitor, showed a similar effect. Both treatments, quercetin and Fer-1, reduce Kim-1 (a biomarker of renal tubular injury) and the production of ROS when compared with the HG treatment (Figure 1). Using RNA sequencing, this group also found that HG induces an abnormal activation of the ferroptosis pathway, which was confirmed via a decrease in GSH and increases in iron, MDA, and 4-HNE contents. In addition, an ultrastructural analysis conducted via transmission electron microscopy (TEM) showed the reduction in mitochondrial crista and even disappearance and outer membrane rupture in HK-2 cells. These mitochondrial alterations were alleviated with quercetin and Fer-1 treatments. Also, these researchers found that GPX4, ferritin heavy polypeptide 1 (FTH-1), and solute carrier family 7-member 11 (SLC7A11) mRNA levels decrease in HK-2 cells treated with HG but are restored via quercetin and Fer-1 treatment. These effects were similar in type 2 diabetic mice with kidney injury, where quercetin decreased the injury by decreasing ferroptosis. Moreover, quercetin activates nuclear factor erythroid 2-related factor 2 (Nrf2), inducing the expression of its target genes such as heme-oxygenase (HO-1) both in mice and HK-2 cells, concluding that quercetin alleviates renal injury via the Nrf2/heme oxigenase (HO-1) signaling pathway, which inhibits ferroptosis cell death [7] (Figure 1). Quercetin also decreases erastin-induced ferroptosis in HK2 and NRK-52E and ferroptosis induced by ischemia/reperfusion (I/R) and folic acid (FA) during acute kidney injury (AKI). That is, quercetin augments cell viability and GSH in these linear cellular models, decreasing MDA and lipid ROS. Regarding IR and FA mice models, quercetin reduces blood urea nitrogen (BUN) and the creatinine markers of kidney injury. Quercetin also reduced MDA content but increased the GSH level in the kidney of both AKI models (Figure 1). The increase in GSH was associated with the induction of the glutathione pathway metabolism via quercetin, where quercetin upregulates SLC7A11 and solute carrier family 3 member 2 (SLC3A2) levels but downregulates the activating transcription factor 3 (ATF3) and HO-1 levels (Figure 1). The investigation explored if ATF3 silencing avoids ferroptosis, and indeed, this silencing induces the augmentation of GPX4 and SLC7A11, which was related to the decrease in ROS lipids in in vivo and in vitro models. Interestingly, this research also showed that ferroptosis recruits macrophages via chemokine (C-C motif) ligand 2 (CCL2) chemokine, inducing a pro-inflammatory state, but quercetin inhibits ferroptosis-induced inflammation in vitro (Figure 1). This effect also occurs in in vivo models, where the infiltration of macrophages and pro-inflammatory cytokines such as tumor necrosis factor α (TNF-α), interleukin (IL)-1β, and IL-6 were increased in IR-induced AKI and FA-induced AKI mice models but was mitigated after quercetin and Fer-1 administration [8]. Thus, this study provides evidence that quercetin reduces ATF3 expression, downregulating the ferroptosis downstream signaling pathway, inhibiting the recruitment of macrophages, and avoiding inflammation and AKI.

## 4. Quercetin Avoids Ferroptosis in Liver Injury

**Figure 2:**
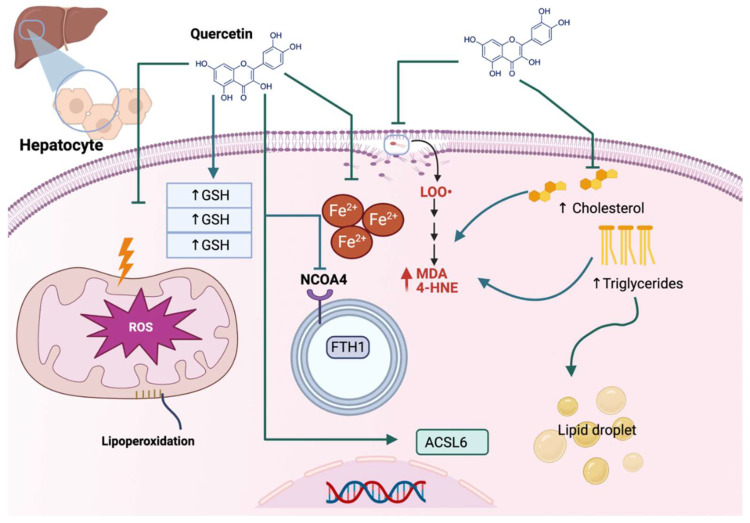
Quercetin alleviates ferroptosis in liver injury. In hepatocytes, quercetin prevents reactive oxygen species (ROS) overproduction in mitochondria, avoiding lipoperoxidation, increasing glutathione (GSH), and avoiding iron (Fe) accumulation. Quercetin reacts with the autophagic cargo receptor nuclear receptor coactivator 4 (NCOA4), blocking the degradation of iron storage protein ferritin heavy chain 1 (FTH1) and, thus, preventing Fe accumulation. Moreover, quercetin avoids lipids accumulation (↑ cholesterol and triglycerides), which blocks the formation of malondialdehyde (↑ MDA), 4-hydroxynonenal (↑ 4-HNE), and lipid droplets. Quercetin also upregulates the transcription of acyl-CoA synthetase long-chain family member 6 (ACSL6), which prevents ferroptosis.

Non-alcoholic fatty liver disease (NAFLD) is related to hepatic steatosis, a cell condition characterized by excess triglyceride accumulation stored as lipid droplets in the cytosol of hepatocytes, which may develop into nonalcoholic steatohepatitis (NASH), fibrosis, cirrhosis, and hepatocellular carcinoma [9]. NAFLD also is characterized by oxidative stress and lipid peroxidation, so inhibiting oxidative stress and lipid peroxidation can be an effective NAFLD treatment strategy [10]. Quercetin has beneficial effects on a high-fat diet (HFD)-induced NAFLD, inhibiting ferroptosis. The latter was demonstrated by Jiang et al., who found that quercetin reduced the total triglycerides and total cholesterol in the serum and liver together with a reduction in mitochondria ROS (MtROS), lipid peroxidation, and liver iron content, increasing GPX4 and GSH/glutathione disulfide (GSSG) ratio (Figure 2). Quercetin also decreases ROS and lipid droplet accumulation in steatotic L-02 cells. Thus, quercetin reduces ROS, lipid peroxide, and iron overload, inhibiting ferroptosis in hepatocytes and livers of HFD-fed mice [11]. 

Acrylamide (ACR), produced in carbohydrate-rich foods during thermal processing, can also generate hepatotoxicity. During this hepatotoxicity, ACR elevates the levels of alanine transaminase (ALT), aspartate aminotransferase (AST), ROS, lipid peroxidation, and ferroptosis, which are alleviated via quercetin. Huang et al. [12] demonstrated that the mechanism of quercetin in the reduction of ferroptosis, both in vivo and in vitro, involves the inhibition of ROS production and MDA content and the increase in GPX4 and GSH protein levels. Moreover, this group demonstrated that quercetin downregulates the ferroptosis-promoting genes like acyl-CoA synthetase long-chain family member 6 (ACSL6) but upregulates the ferroptosis-inhibiting genes such as SLC7A11. Quercetin also inhibits the autophagic cargo receptor nuclear receptor coactivator 4 (NCOA4), blocking the degradation of iron storage protein ferritin heavy chain 1 (FTH1), which decreases the intracellular iron levels and the consequent ferroptosis (Figure 2). The above works show the anti-ferroptosis effect of quercetin in hepatoxicity induced via ACR and excess triglyceride accumulation of lipids, which are associated with increased ROS, oxidative stress, and lipid peroxidation.

## 5. Quercetin Avoids Ferroptosis in Central Nervous System Injury

**Figure 3:**
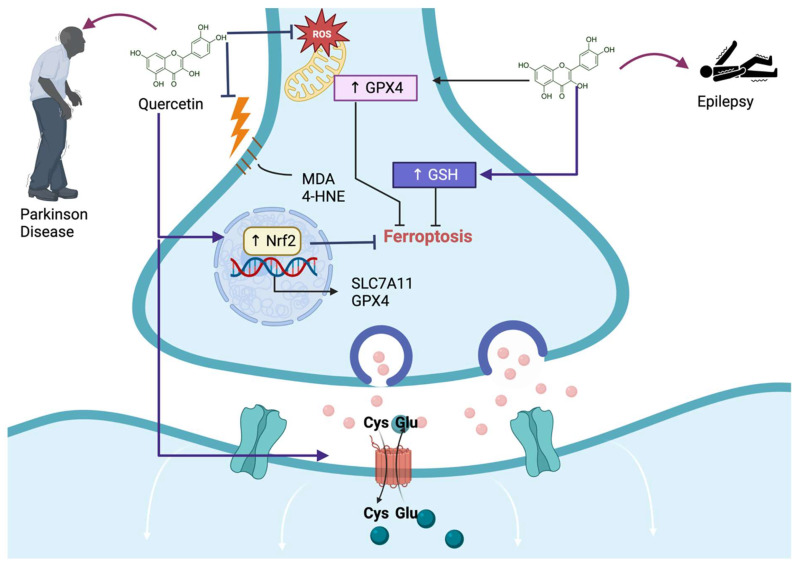
Quercetin alleviates central nervous system injury induced via ferroptosis. Quercetin avoids Parkinson’s disease and epilepsy by preventing reactive oxygen species (ROS) production in mitochondria, avoiding malondialdehyde (MDA) and 4-hydroxynonenal (4-HNE) formation, increasing glutathione peroxidase 4 (GPX4) and glutathione (GSH). These mechanisms prevent ferroptosis, alleviating these diseases. Additionally, quercetin-induced ferroptosis by activating the nuclear factor erythroid 2-related factor (↑ Nrf2) induces the transcription of solute carrier family 7-member 11 (SCLC7A11) and GPX4. Moreover, quercetin induces the activation of SCLC7A11, preventing ferroptosis.

Parkinson’s disease (PD) is a progressive neurodegenerative disorder of nigrostriatal dopaminergic (DA) neurons; until now, a disease without a cure, where treatments can only help alleviate symptoms. Therefore, it is urgent to develop effective drugs for treating PD. Since PD is related to oxidative stress and damage induced via lipid peroxidation, causing cell death, such as ferroptosis of DA neurons, compounds such as quercetin can alleviate OS, preventing the development of ferroptosis and PD or stopping the progression of the disease. Jiang et al. [13] demonstrated that quercetin decreases ferroptosis-induced cell death via the cytotoxic compound 1-methyl-4-phenylpyridinium (MPP+) in the neuroblastoma cell line SH-SY5Y. This group of researchers showed that quercetin decreases MDA, iron content, and NCOA4 levels, increasing mitochondrial membrane potential, GPX4, and SLC7A11 levels (Figure 3). Since Nrf2 induces the expression of the last two proteins, the authors measured the levels of this transcription factor in this investigation, finding that quercetin increases Nrf2 levels after MPP+ had decreased its levels to induce ferroptosis. Thus, this research found that quercetin alleviates ferroptosis in a neuroblastoma cell line influenced by MPP+ via Nrf2 pathway activation.

Epilepsy is a neurological disorder where classical antiepileptic drugs induce drug toxicity and cognitive function impairment, which makes it essential to develop novel therapies to solve this issue. It has been suggested that the activation of Nrf2 could reduce ferroptosis cell death, representing a potential therapy for seizures. Quercetin, as a natural polyphenol that activates Nrf2, could avoid ferroptosis, alleviate seizure-induced neuron death, and preserve cognitive function. Xie et al. [14] found that in a kainic acid-induced epileptic mouse model, quercetin exerts protective effects on seizure-induced neuronal death in an epileptic mouse model. They even found that quercetin protects against glutamate-induced cell death in HT22, a mouse hippocampus of immortalized neuronal cell line. This group found that quercetin activated Nrf2, augmenting GSH and decreasing MDA and 4HNE, preventing ferroptosis and alleviating seizure-like behaviors and cognitive decline in kainic acid-induced epileptic mice (Figure 3). Regarding HT22 neuronal cell death induced via glutamate, this group found that quercetin exerts neuroprotective effects by activating Nrf2, which activates the antioxidant system augmenting GSH, which is related to the reduction of lipid ROS production, MDA, and 4HNE levels, thereby decreasing cell death through the ferroptosis pathway (Figure 3). They also found that quercetin activates the silent mating type information regulation 2 homolog 1 (SIRT1)/Nrf2/SLC7A11/GPX4 pathway as a crucial pathway to prevent glutamate-induced ferroptosis in HT22 cells [14]. Therefore, the authors suggested that quercetin protects against seizure-induced neuron death in vivo and in vitro, alleviating cognitive function impairment by activating SIRT1/Nrf2/SLC7A11/GPX4 pathway.

Spinal cord injury (SCI) produces impaired mobility, sensory, and autonomic dysfunctions. Oligodendrocyte progenitor cells (OPCs) differentiate into mature oligodendrocytes, and remyelinate damaged axons. The loss of OPCs results in poor SCI recovery, so inhibiting OPC loss is challenging to overcome. It has been shown that quercetin prevents the ferroptosis of OPCs by downregulating the inhibitor of DNA binding 2 (Id2)/transferrin pathway. In this work, the authors demonstrated that erastin increases OPCs cell iron concentration, ROS generation, and prostaglandin-endoperoxide synthase 2 (PTGS2) protein levels, decreasing cell viability, GSH, and GPX4 protein levels. However, quercetin increases cell viability, GSH, and GPX4 levels, ameliorating iron concentration, ROS production, and PTGS2 levels. Moreover, erastin also induces shrunken mitochondria, increased membrane density, and decreased mitochondrial cristae, alleviated with quercetin treatment. These effects induced quercetin to reduce axonal and myelin loss caused by erastin treatment [15]. Interestingly, the overexpression of transferrin reversed the antiferroptosis quercetin protective effect and even reversed the quercetin protective effect of axonal and myelin loss. The transferrin gene has a non-palindrome E-box sequence (CATCTG) with which Id2 interacts. When Id2 was overexpressed, Id2 abolished the quercetin protective effect on OPC ferroptosis, increasing transferrin expression, iron concentration, ROS production, and abnormally structured mitochondria and decreasing GSH and cell viability [15]. It also was shown that in an in vivo SCI model, the protein levels of Id2 and transferrin are significantly higher, but quercetin treatment significantly reduced the expression of both proteins, decreasing iron content. Furthermore, quercetin treatment increases GSH levels, GPX4 gene expression, and protein levels and even significantly decreases PTGS2 levels [15]. Thus, these pieces of evidence demonstrate that quercetin ameliorated ferroptosis in spinal cord tissue, identifying quercetin as a negative regulator of ferroptosis in OPC and SCI, inducing myelin and axonal generation by downregulating the pathway Id2/transferrin.

## 6. Quercetin Prevents Ferroptosis in Bone Marrow-Derived Mesenchymal Stem Cells (BMSCs) Injury

**Figure 4:**
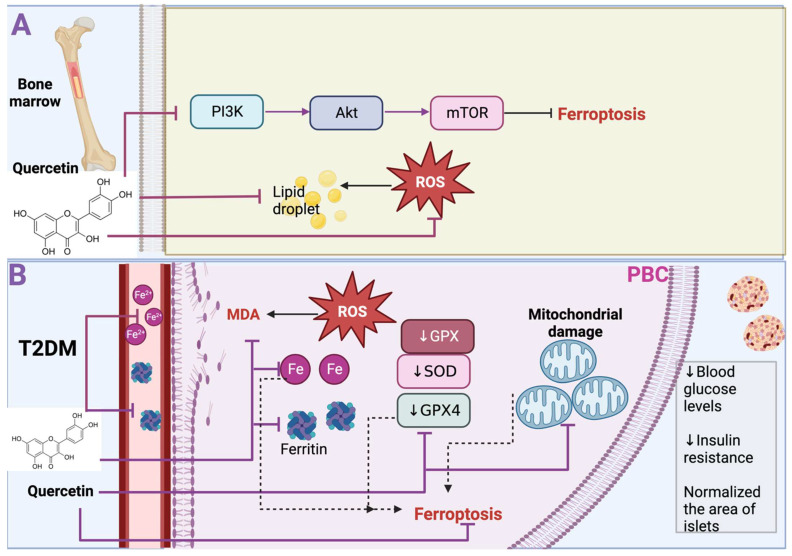
Quercetin avoids ferroptosis in bone marrow-derived mesenchymal stem cells (BMSCs) and type 2 diabetes mellitus. (**A**) In BMSCs, quercetin inhibits phosphatidylinositol 3-kinase (PI3K)/Protein kinase B (AKT)/mammalian target of rapamycin (mTOR) signaling pathway, preventing ferroptosis. Furthermore, quercetin prevents ferroptosis by inhibiting lipid droplets and producing reactive oxygen species (ROS). (**B**) In the blood of T2DM, quercetin prevents iron (Fe) and ferritin accumulation. In pancreatic β cells, quercetin prevents increased Fe and ferritin, preventing ROS and malondialdehyde (MDA) content increase. Quercetin also prevents mitochondrial damage and the reduction in glutathione peroxidase (↓ GPX), superoxide dismutase (↓ SOD), and glutathione peroxidase 4 (↓ GPX4), avoiding ferroptosis. Black arrows indicate induce; inhibitory arrows indicate prevent.

Osteoporosis is a disease that increases the risk of osteoporotic fracture and is characterized by low bone mineral density. Osteoporosis is caused by excessive bone resorption or inadequate new bone formation during bone remodeling [16]. This bone remodeling is related to bone metabolism, closely related to oxidative stress. For instance, high ROS levels induce osteoclastogenesis and stimulate osteoclast activity, leading to bone loss [17]. Oxidative stress leads to cell death via ferroptosis in osteoporosis, which the antioxidant properties of quercetin can attenuate. In fact, this has been demonstrated in BMSCs under oxidative stress induced via hydrogen peroxide (H_2_O_2_). H_2_O_2_ treatment induces ROS production, reducing BMSC viability and osteogenic key proteins such as runt-related transcription factor 2 (RUNX2), alkaline phosphatase (ALP), and osteopontin (OPN) (Figure 4). However, quercetin treatment promotes BMSC proliferation, reduces ROS production, and promotes ALP and OPN levels, which results in the osteogenic differentiation of BMSCs. Moreover, ferroptosis-related markers such as GPX4, SLC7A11, and Nrf2 are increased with quercetin treatment, concluding that quercetin reduces H_2_O_2_-induced ferroptosis in BMSCs. Quercetin also inhibits the phosphatidylinositol 3-kinase (PI3K)/Protein kinase B (AKT)/mammalian target of rapamycin (mTOR) signaling pathway. Thus, quercetin inhibited ferroptosis and the dephosphorylation levels of PI3K, AKT, and mTOR, maintaining the viability and the osteoblastic differentiation of BMSCs upon H_2_O_2_ stimulation [18] (Figure 4A). Note that quercetin, after reducing ROS with its antioxidant properties, is oxidized and metabolized to quercetin Diels–Alder anti-dimer (QDAD), a molecule that presents seven phenolic -OHs, two phenolic -OHs more than quercetin. It has been demonstrated that phenolic -OHs enhance the antioxidant and antichelating effect, but QDAD demonstrated that this product does not have a bigger antioxidant and antichelating effect. For example, although quercetin and QDAD inhibited lipid ROS and ROS production and decreased ferroptosis cellular death in BMSCs induced via erastin, quercetin was more effective than QDAD in reducing ferroptosis as quercetin significantly decreased ROS lipids and ROS production and augmented more cell viability in comparison with QDAD [19]. It has been suggested that QDAD is difficult to oxidize as it resembles orthoquinone quercetin, which has a weak antioxidant effect. Therefore, after metabolizing quercetin to QDAD, it no longer has an antiferroptosis impact. The above works demonstrated that quercetin reduces ferroptosis in BMSCs cells and that metabolites such as QDAD are not more antiferroptotic compounds, which suggested that it is crucial to avoid oxidizing metabolites before using quercetin.

## 7. Quercetin Alleviates Ferroptosis in Pancreatic β Cells Injury in Type 2 Diabetes (T2DM)

During T2DM, iron deposition has been found in pancreatic β cells (PBC), which suggests that ferroptosis could contribute to the dysfunction of PBC. Indeed, it has been shown that in a model of T2DM induced via a high fructose diet (HFD), the iron level in serum significantly increases, and even ferritin light chain levels were significantly increased in PBC in the diabetic group. Interestingly quercetin treatment decreases the iron level in serum and the levels of ferritin in PBC. Moreover, in the T2DM model, GPX levels, SOD activity, and GSH significantly decreased, increasing ROS production and MDA content. In contrast, quercetin treatment reduces ROS levels and MDA content by augmenting GPX4 and GSH levels and SOD activity (Figure 4B). Additionally, the T2DM model shows smaller mitochondria with increased membrane density, a feature of mitochondrial damage, which is a characteristic of ferroptosis. Even diabetic mice displayed fewer mitochondria, with shrunken accompanied by progressive loss of cristae. Interestingly these effects were alleviated via quercetin [20]. The latter results demonstrated that the T2DM model induced via HFD presents ferroptosis cell death, and quercetin prevents this effect, which causes lowered blood glucose levels, restores homeostasis model assessment (HOMA)-β index, and decreases insulin resistance in T2DM. Furthermore, quercetin normalized the area of islets, their number, and their perimeter, having a protective effect on the size and structure of the islet and mitigating their disorganization. This effect increased insulin in diabetic mice [20]. Thus, this work demonstrated that high glucose in mice induces ferroptosis cell death in PBC, contributing to cell dysfunction; however, quercetin treatment inhibits this cell death.

## 8. Quercetin Alleviates Ferroptosis during Inflammation

Ferroptosis is also present in pathological processes such as lung inflammation and asthma. Wang et al. [21] demonstrated that quercetin alleviates ferroptosis in lipopolysaccharide (LPS)/ovalbumin (OVA)-induced neutrophilic asthma mouse model. This neutrophilic airway inflammation of the LPS/OVA-induced model presents high 4HNE levels in lung tissue, an increase in MDA levels both in serum and in lung tissue accompanied by a decrease in GPX4 and SLC7A11 levels and mitochondria that are severely distorted and enlarged. Interestingly, quercetin reduces 4HNE and MDA levels, increasing GPX4 and SLC7A11 and restoring mitochondria. This anti-ferroptosis effect of quercetin was associated with the alleviation of the neutrophilic airway inflammation in the neutrophilic asthma mouse model, where quercetin decreases the levels of inflammatory cytokines such as TNF-α, IL-6, IL-1β, and IL-17A in lung tissue and decrease inflammation scores, neutrophil cell counts and chemokine (C-X-C motif) ligand 1 (CXCL1, a chemokine that recruits and activates neutrophils) in bronchoalveolar lavage fluids of this inflammation model [21]. This group of researchers also found that quercetin regulates M1 macrophage polarization. This finding was found in primary bone marrow-derived macrophages (BMDMs), where M0 macrophages were differentiated into M1 macrophages via the stimulation of LPS and IFN-γ. Interestingly, quercetin significantly decreased the proportion of the mRNA expressions of inducible nitric oxide synthase (iNOS) and the cluster of differentiation (CD)86 (M1 markers) in M1 macrophages, avoiding M0 macrophages differentiation into M1. Furthermore, this research group found that quercetin decreased the mRNA expressions of M2 macrophage markers such as CD206 and Arg1 in macrophages where the differentiation of M0 macrophages into M2 macrophages was induced by stimulating IL-4 and IL-13 [21]. Thus, these findings proved that quercetin suppressed ferroptosis and macrophage polarization associated with neutrophilic airway inflammation.

The above evidence suggests that quercetin is a promising ferroptosis inhibitor in renal injury, liver, central nervous system, pancreatic β cells, BMSCs, and airway inflammation; however, more investigation is necessary about the side effects of quercetin in healthy tissues.

## 9. Quercetin Promotes Ferroptosis as Anticancer Activity

**Figure 5:**
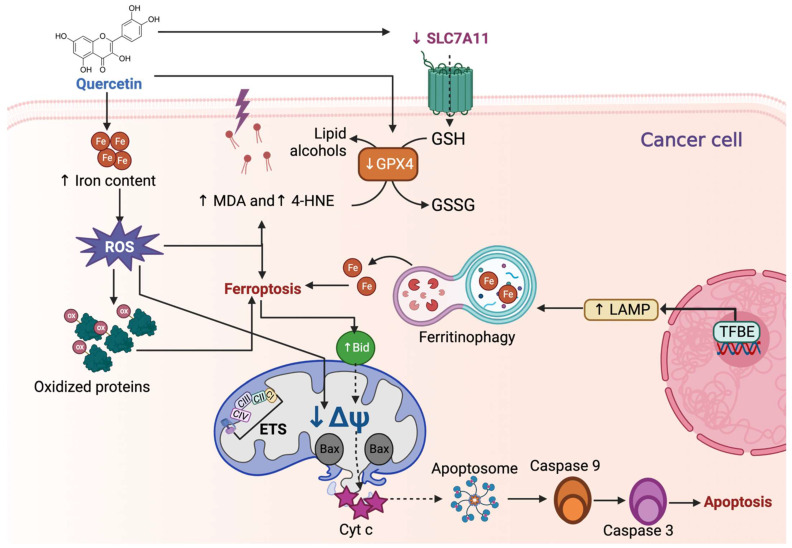
Quercetin induces ferroptosis in cancer cells. Quercetin increases the iron (Fe) content, which promotes reactive oxygen species (ROS) overproduction, influencing lipoperoxidation by increasing malondialdehyde (↑ MDA) and 4-hydroxynonenal (↑ 4-HNE). Additionally, ROS induces the oxidation of proteins and the decrease in mitochondrial membrane potential (↓ΔΨ), and along with ↑ MDA and ↑ 4-HNE, activates ferroptosis. Quercetin-induced ferroptosis by reducing glutathione peroxidase 4 and solute carrier family 7-member 11 (↓ SLC7A11). The decrease in SLC7A11 is also attributed to p53. Quercetin also induces ferroptosis by inducing ferritinophagy, a specialized autophagy mechanism in which Fe is recycled. Ferritinophagy is caused by transcription factor EB (TFEB), which generates the expression of lysosomal-associated membrane protein 1 (LAMP-1), involved in the first steps of autophagy. Ferroptosis can induce apoptosis by upregulating Bid (↑ Bid) protein, which ↓ΔΨ, causing the release of cytochrome c (Cyt c). Cyt c release induces apoptosome formation, activating caspase 9 and 3, inducing apoptosis. CI: complex I; CII: complex II; CIII: complex III; CIV: complex IV; ETS: electron transfer system.

Quercetin shows several anticancer activities such as cell cycle arrest, antiproliferative and antiangiogenic activities, autophagy, the induction of intrinsic and extrinsic apoptotic pathways, and antimetastatic effects in an extensive range of cancers, including lung, ovarian, prostate, breast, colorectal, and bladder cancers [8,9]. Although the mechanism of action of quercetin as an anticancer compound is unclear, death from ferroptosis could be a target during anticancer therapy [11]. The latter is because cancer cells are more iron-dependent and more sensitive to ferroptosis than normal cells [12]. For instance, it has been demonstrated that 10 μM of quercetin significantly decreases cell viability in MCF-7 and MDA-MB-231 breast cancer cells. This decrease was similar to the effect induced via the inductor of ferroptosis erastin. Quercetin, like erastin, also significantly augments iron concentration, MDA, and carbonyl protein levels, demonstrating the induction of ferroptosis [22]. Quercetin also decreases the proliferation and cell migration of HEC-1-A endometrial carcinoma cells, where quercetin increases ROS production and transferrin receptors and reduces mitochondria membrane potential, aconitase 1 (an iron regulatory protein also known as iron-responsive element-binding protein 1, IREB1), GPX4, and SLC7A11 levels [23] (Figure 5). The increase in ferroptosis markers demonstrates that quercetin induces ferroptosis as a cell death in HEC-1-A. Since the authors also found an increase in apoptosis in these cells when HEC-1-A were treated with quercetin, they suggested that this compound activates ferroptosis via apoptosis. The activation of ferroptosis might be attributed to p53, which inhibits SLC7A11 transcription, leading to cellular ferroptosis sensitivity. However, in HEC-1-A, p53 expression did not change, suggesting that other cell signaling pathways can activate ferroptosis or that apoptosis can activate p53-independent ferroptosis [23]. 

Ferroptosis is induced via autophagy processes such as ferritinophagy, a lysosomal pathway that promotes ferritin degradation, inducing iron ions release (Figure 5) [14]. Lysosomes are regulated in a transcription-dependent way via transcription factor EB (TFEB), which facilitates gene expressions related to the regulation of lysosomal function [15]. The activation of lysosomes to degrade ferritin may be a potential anticancer mechanism, so one of the mechanisms by which quercetin may induce ferroptosis is TFEB activation. This was demonstrated by An and Hu [22] when they observed that 10 μM of quercetin induces a significant translocation and activation of TFEB from 4 to 24 h of treatment with quercetin. TFEB activation induces the transcription of lysosomal-associated membrane protein 1 (LAMP-1), one of the lysosome-related proteins transcriptionally regulated via TFEB (Figure 5). Therefore, when quercetin activates TFEB in breast cancer cells, it activates ferritinophagy via LAMP, degrading ferritin and promoting iron release by promoting ferroptosis as a cancer cell death mechanism. This effect also was demonstrated by Wang et al. [24], who show that quercetin induces TFEB activation, ferritinophagy, iron release, and ferroptosis promotion. This group showed that quercetin decreases cell viability and induces p53-independent cell death of human HepG2 and Hep3B hepatocellular carcinoma cells, MDA-MB-231 breast cancer cell line, HCT116 colorectal cancer cells, and HeLa cervical cancer cells [24]. In these cell lines, quercetin promotes lysosome activation via TFEB activation, activating LAMP1 and vacuolar-type ATPase (V-ATPase) subunits and promoting ferritin degradation, iron release, ROS production, and lipid peroxidation increase, which induces cell death via ferroptosis. Interestingly, ferroptosis induces Bid (proapoptotic protein) and caspase 9 activation and PARP cleavage, promoting apoptosis. This suggests that ferroptosis may act upstream of apoptosis, and Bid is a critical mediator of the connection between ferroptosis and apoptosis [24].

Quercetin also works as an inducer of ferroptosis oppositely, as mentioned in the first part of this review, this double edge of quercetin’s mechanism of action warns us about its use, highlighting the search for the therapeutic concentration of this compound. Finding this window for quercetin treatment in each disease will provide us with a powerful tool for using quercetin as a potential anticancer compound or alleviating illnesses associated with the liver, pancreas, kidney, central nervous system injury, or inflammation. It also provided information on whether this concentration could be toxic to other healthy cells, which warrants further investigation. We present in Table 1 an overview of quercetin’s effects on the diseases presented in this review.

## 10. Conclusions and Remarks

**Figure 6:**
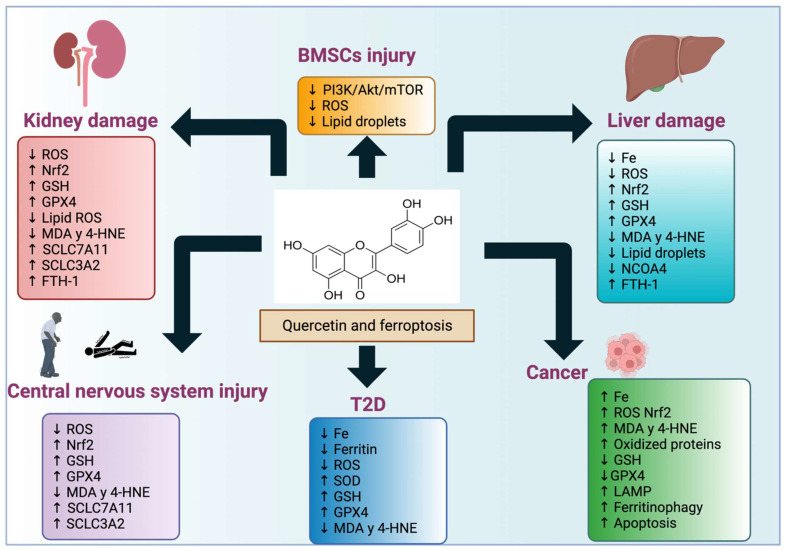
Quercetin effects in ferroptosis-induced diseases. Quercetin affects the induction or blocking of ferroptosis in the conditions or disorders mentioned in this review.

Quercetin has several pharmacological effects as a form of anti-cancer and cardiovascular protection due to its chelating, antioxidant, and anti-inflammatory properties. Moreover, quercetin has been shown to inhibit ferroptosis and improve renal damage, type 2 diabetes, inflammation, and the liver of mice fed a high-fat diet. Therefore, quercetin has the potential as a broad therapeutic compound and could be helpful to treat ferroptosis in many diseases where it is desired to prevent this death or induce ferroptosis cell death in conditions such as cancer. However, the potential application of quercetin in treating these diseases still needs further investigation since the possible toxic effect that this component could have on patients must be ruled out. Figure 6 summarizes the impact that quercetin has on the induction or blocking of ferroptosis in the different diseases or disorders mentioned in this review.

## Figures and Tables

**Table 1:** Induction of ferroptosis and its relief in different diseases or disorders.

Disease or Disorder	Induction of Ferroptosis	Effect of Quercetin in Ferroptosis	Reference
Cell injury of human kidney proximal tubular epithelial (HK-2) via HG treatment	↓ GSH ↑ iron, MDA, and 4-HNE content ↓ mitochondrial crista and even disappearance and outer membrane rupture in HK-2 cells. ↓ GPX4, FTH-1, SLC7A11 mRNA levels	↑ cell viability ↓ Kim-1 and ROS production ↓ iron, MDA, and 4-HNE content ↑ mitochondrial crista ↑ Nrf2 and HO-1 **↓ ferroptosis**	[7]
Ferroptosis induced via erastin in renal tubular cell lines HK2 and NRK-52E and ferroptosis induced via I/R and FA during AKI	Recruits macrophages via CCL2 chemokine	↑ cell viability and GSH ↓ MDA and lipid ROS in HK2 cells In IR and FA mice models, quercetin ↓ BUN and creatinine markers of kidney injury ↑ GPX4, SLC7A11 and SLC3A2 ↓ TNF-α, IL-1α, IL-6, and ATF3 expression, and the recruitment of macrophages, avoiding inflammation, and AKI **↓ ferroptosis**	[8]
NAFLD is characterized by ferroptosis	↑ Oxidative stress and lipid peroxidation	↓ Total triglycerides and cholesterol in serum and liver, MtROS, lipid peroxidation, and liver iron content ↑ GPX4 and GSH/GSSG ratio **↓ ferroptosis**	[11]
Hepatotoxicity via ACR ↑ ALT, AST, and ferroptosis	↑ ROS and lipid peroxidation	↓ ROS, MDA, ferroptosis-promoting genes such as ACSL6 ↑ GPX4, GSH levels, and ferroptosis-inhibiting genes such as SLC7A11. Quercetin reacts with autophagic cargo receptor NCOA4, ↓ iron degradation storage in FTH1 **↓ ferroptosis**	[12]
Ferroptosis induced via MPP+ in a neuroblastoma cell line	↑ OS and lipid peroxidation	↓ MDA, iron content, and NCOA4 levels ↑ mitochondrial membrane potential, GPX4, SLC7A11 and Nrf2 pathway **↓ ferroptosis**	[13]
Seizure-induced neuronal death via ferroptosis in a kainic acid-induced epileptic mouse model HT22 neuronal cell death induced via glutamate	**↑ ferroptosis**	↑ Nrf2 and GSH ↓ MDA and 4HNE ↓ lipid ROS ↑ SIRT1/Nrf2/SLC7A11/GPX4 pathway **↓ ferroptosis**	[14]
The loos of OPCs results in poor SCI recovery	↑ Iron concentration, ROS generation, and PTGS2 protein levels ↓ cell viability, GSH, and GPX4 protein levels ↑ shrunken mitochondria, membrane density ↓ decreased mitochondrial cristae	↓ DNA binding 2 (Id2)/transferrin pathway, iron concentration, ROS production, and PTGS2 levels ↑ cell viability, GSH, and GPx4 levels **↓ ferroptosis** ↑ transferrin and Id2 reversed the protective effect against ferroptosis and axonal and myelin loss induced via quercetin, inducing ↑ iron concentration, ROS production, and abnormally structured mitochondria, decreasing GSH and cell viability	[15]
Oxidative stress leads to cell death via ferroptosis in osteoporosis	↑ ROS production ↓ BMSCs viability	↑ BMSCs proliferation, GPX4, SLC7A11, and Nrf2 ↓ ROS production **↓ ferroptosis**	[18]
Iron deposition has been found in PBC, inducing ferroptosis and dysfunction of PBC, during T2DM.	↓ GPX levels, SOD activity, and GSH ↑ ROS production and MDA content Smaller, fewer, and shrunken mitochondria with increased membrane density, accompanied by progressive loss of cristae.	↑ GPX4 levels, SOD activity, and GSH levels ↓ ROS levels and MDA ↓ Mitochondria dysfunction **↓ ferroptosis**	[20]
Ferroptosis is induced in LPS/OVA neutrophilic asthma mouse model	↑ 4HNE, MDA, and mitochondria distorted and enlarged ↓ GPX4 and SLC7A11 levels	↓ 4HNE, MDA, and mitochondria dysfunction ↑ GPX4 and SLC7A11 levels **↓ ferroptosis**	[21]
↓ Cell viability of MCF-7, MDA-MB-231 breast cancer		↑ Iron concentration, MDA, carbonyl protein levels, TFEB, and LAMP proteins **↑ ferroptosis**	[22]
↓ Proliferation and cell migration of HEC-1-A endometrial carcinoma cells.		↑ ROS production and transferrin receptor ↓ mitochondria membrane potential, aconitase 1, GPX4, and SLC7A11 levels **↑ ferroptosis**	[23]
↓ Cell viability of human hepatocellular carcinoma cells HepG2 and Hep3B, colorectal cancer cells HCT116, and cervical cancer cells HeLa		↑ TFEB activation, activating LAMP1, V-ATPase, ferritin degradation, iron release, ROS production, and lipid peroxidation increase **↑ ferroptosis** ↑ Bid, caspase 9, and PARP cleavage promoting apoptosis, suggesting that ferroptosis may act upstream of apoptosis	[24]

Ferritin heavy polypeptide 1 (FTH-1), solute carrier family 7-member 11 (SLC7A11), nuclear factor erythroid 2-related factor 2 (Nrf2), hemoxygenase (HO-1), ischemia/reperfusion (I/R), folic acid (FA), acute kidney injury (AKI), blood urea nitrogen (BUN), solute carrier family 3 member 2 (SLC3A2), tumor necrosis factor α (TNF-α), interleukin (IL), chemokine (C-C motif) ligand 2 (CCL2) chemokine, mitochondria ROS (MtROS), glutathione disulfide (GSSG), non-alcoholic fatty liver disease (NAFLD), acrylamide (ACR), alanine transaminase (ALT), aspartate aminotransferase (AST), reactive oxygen species (ROS), acyl-CoA synthetase long-chain family member 6 (ACSL6), nuclear receptor coactivator 4 (NCOA4), protein ferritin heavy chain 1 (FTH1), high glucose (HG), 1-methyl-4-phenylpyridinium (MPP+), oxidative stress (OS), silent mating type information regulation 2 homolog 1 (SIRT1), prostaglandin-endoperoxide synthase 2 (PTGS2), bone marrow-derived mesenchymal stem cells (BMSCs), pancreatic β cells (PBC), type 2 diabetes (T2DM), lipopolysaccharide (LPS)/ovalbumin (OVA), transcription factor EB (TFEB), vacuolar-type ATPase (V-ATPase), lysosomal-associated membrane protein 1 (LAMP-1).

## Data Availability

Not applicable.
